# Editorial: Integrative root responses to multiple environmental signals for plant resilience

**DOI:** 10.3389/fpls.2025.1744037

**Published:** 2026-01-14

**Authors:** Jose Sebastian, Masashi Yamada, María José García, Yutaka Miyazawa

**Affiliations:** 1 Department of Biological Sciences, Indian Institute of Science Education and Research, Berhampur (IISER Berhampur), Odisha, India; 2 Agricultural Biotechnology Research Center, Academia Sinica, Taipei, Taiwan; 3 Biotechnology Center in Southern Taiwan, Academia Sinica, Tainan, Taiwan; 4 Department of Agronomy (DAUCO-María de Maeztu Unit of Excellence), Edificio Celestino Mutis (C-4), Universidad de Córdoba, Córdoba, Spain; 5 Faculty of Science, Yamagata University, Yamagata-shi, Japan

**Keywords:** climate resilience, root plasticity, root system architecture, global warming, root traits, plant stress tolerance

## Root systems and plant resilience

The progressive increase in the Earth’s average surface temperature, commonly referred to as global warming, poses a significant threat to global food security. Global warming can fuel frequent heatwaves, severe droughts, and other extreme weather events worldwide. Plants are particularly susceptible to these weather extremes, which can cause poor growth, lower photosynthetic efficiency, increased vulnerability to pathogens etc., leading to reduced crop yields (Lesk et al., 2022; Seth and Sebastian, 2024; Rhee et al., 2025). The root system plays a central role in plant resilience attributes through its role in water and nutrient absorption, as well as its intricate partnerships with microorganisms (Koevoets et al., 2016; Lynch, 2021; Lombardi et al., 2021; Yetgin, 2024; Ramachandran et al., 2025). Root system traits, including anatomical features, phenotypic plasticity, physiological adaptations, and architecture, can all influence a plant’s ability to acclimate and survive under challenging environmental conditions. It has been documented that improvements in the root system characteristics such as anatomy (larger cortical aerenchyma, increase in cortical cell size, metaxylem morphology etc.), biomass (dense and vigorous root system with continuous development), architecture (a robust deep rooting system), growth rate, etc., significantly influence overall plant performance and enable plants to handle suboptimal environmental conditions (Lynch, 2022; Yetgin, 2024). Thus, a robust root system’s plasticity can significantly enhance a plant’s ability to survive, adapt, and thrive under challenging conditions by modulating root traits, which permits effective exploration of the soil and maximizes water/nutrient uptake.

## Integrative root responses to environmental signals

Various environmental factors, including the availability of water, nutrients, temperature, soil density, and physicochemical properties, impact root system attributes by influencing metabolism, physiology, and development (Yetgin, 2024). These root-environment interactions often lead to modifications in root system characteristics, such as growth pattern, morphology, length, density, depth, and architecture (Ramachandran et al., 2025). Phytohormones play a key role in this process by acting as integrators of external signals (such as soil moisture levels, temperature, and pathogens) with the internal developmental program, coordinating root growth and branching patterns (Ghanem et al., 2011). These adaptive modifications help the plant to optimize resource capture and survival under subpar growth conditions (Callaway and Li, 2020; Karlova et al., 2021; Yetgin, 2024; Ramachandran et al., 2025). In general, these external signals are perceived by different cellular mechanisms involving specialized receptors that trigger intracellular signalling pathways leading to changes in gene expression patterns. These transcriptomic changes, in turn, allow the plant to adapt to the changing environment (Ramachandran et al., 2025).

The common root responses to environmental signals can be categorized as tropisms, physiological, anatomical, metabolic, and morphological adaptive changes. For instance, root systems respond to water deficit by directing growth towards sources of moisture (hydrotropism), a deeper, longer, denser root system (alteration in root system architecture), development of specialized structures (root hairs, adventitious roots), increased production of ABA, etc (Kalra et al., 2023). Similarly, to high soil temperature, roots respond by metabolic changes (increase in ROS levels), narrower roots, etc (Seth and Sebastian, 2024). To address nutrient deficiency, roots react by altering their architecture (changing root angle and branching pattern), increasing the production of lateral roots & root hairs, and improving the root-to-shoot ratio, among other responses (Lopez et al., 2023). In response to biotic stress, roots enhance lateral root branching, release specific exudates (to attract beneficial microbes), increase deposition of suberin in cell walls, increase ROS production, alter metabolic processes and hormone production, increase lignification of cell walls, trigger autophagy, etc (Sharma et al., 2023; Chaurasia et al., 2025). Root systems respond to changes in soil properties (mechanical impedance, oxygen deficiency) by increased sloughing of border cells, enhanced exudation to reduce friction, and thickening of the root to provide mechanical strength, etc (Bathke et al., 1992; Bengough et al., 2006; Ogorek et al., 2025). Other features observed in response to mechanical impedance include changes in cell shape (shorter, fatter cells), changes in cell wall composition, etc. The formation of aerenchyma in cortical tissues is a common root response to hypoxia (Ellis et al., 1999; Mustroph et al., 2014; Eysholdt-Derzsó et al., 2024). Exposure to low temperatures inhibits primary root growth and reduces the branching angles between primary and lateral roots, resulting in a deeper rooting system (Nagel et al., 2009). Together, these adaptive root system responses equip plants to establish resilience against unfavourable conditions.

The current Research Topic features five original research articles covering the area of root system responses to multiple environmental signals, focusing on plant resilience. Lin et al. describe the alteration in root exudate metabolome in cotton under progressive drought conditions. The study identified more than 700 unique metabolites induced by drought conditions. Moreover, pathways associated with flavonoid biosynthesis, secondary metabolites, and phytohormone synthesis (ABA and Jasmonic acid), etc., were highly upregulated. Collectively, the study provides valuable insights into drought-induced secondary metabolite modifications in root exudates, which can be used to develop resilience strategies. Song et al. (demonstrate the molecular link between the mitochondrial metabolic pathway and polar auxin transport during root development. Authors describe how an impairment in the TCA cycle affects the internalization and endocytic recycling of PIN- FORMED proteins (PINs), leading to a drastic decline in PIN abundance in the plasma membrane. It appears that enhanced ROS levels, resulting from the disruption of the mitochondrial pyruvate dehydrogenase complex, indirectly contribute to this phenotype. Together, this study reveals how disruptions in cellular energy production affect root growth and development. Zhang et al. investigated the effect of drought stress on *S. miltiorrhiza* seedlings and identified key genes and metabolites related to drought tolerance. Drought affects *S. miltiorrhiza* seedlings primarily by disrupting root growth (enhancing membrane lipid peroxidation and attenuating the antioxidant system) and photosynthesis.

Article by Jiang et al. reports the active trade-offs between the rhizome-system nutrient stress) in six spring wheat genotypes. These researchers identified significant variation in root responses uptake and leaf nutrient resorption (a nutrient conservation mechanism by resorption from senescing leaves), in Moso bamboo (*Phyllostachys edulis*). These nitrogen trade-offs between the root system and leaves appear to be due to the plant’s unique clonal growth characteristics, where it relies heavily on its rhizome for resource storage and translocation between ramets. In summary, the work offers a novel perspective on resource allocation strategies in clonal species, highlighting the importance of coordination between belowground and aboveground processes in nutrient cycling in a cost-effective manner. Finally, Frantova et al. examined the alteration in root morphology induced by drought (early vigor root-growth traits under osmotic(root length and diameter) to drought under low temperature conditions, suggesting inherent variability in root growth regulation among these different genotypes. Taken together, the results showed that specific root traits significantly influence a genotype’s adaptability to drought, emphasizing the importance of integrating both root traits and genetic flexibility into breeding programs to enhance drought tolerance.

Together, these studies advance our understanding of how root systems integrate metabolic, signaling, and resource-allocation strategies to adapt to environmental stress. From drought-induced metabolomic shifts and antioxidant defenses to the interplay between mitochondrial function, auxin transport, and root architecture, and from nutrient trade-offs in clonal species to genotype-specific root traits, the research underscores the multifaceted mechanisms driving root plasticity. Harnessing these insights provides a foundation for developing more resilient crops, offering new routes toward sustainable productivity under increasingly variable environmental conditions.
